# Physicochemical Properties, *in Vitro* Antioxidant Activities and Inhibitory Potential against α-Glucosidase of Polysaccharides from *Ampelopsis grossedentata* Leaves and Stems

**DOI:** 10.3390/molecules16097762

**Published:** 2011-09-09

**Authors:** Yuefei Wang, Xiaoying Bian, Jinhyouch Park, Le Ying, Lisheng Qian, Ping Xu

**Affiliations:** Department of Tea Science, Zhejiang University, Hangzhou 310058, China

**Keywords:** polysaccharides, *Ampelopsis grossedentata*, physicochemical properties, antioxidant activities, α-glucosidase inhibition

## Abstract

In the present study, polysaccharides named ALPS and ASPS were isolated from *Ampelopsis grossedentata* leaves and stems, respectively. Physicochemical properties, *in vitro* antioxidant activities and the inhibitory effects on α-glucosidase of ALPS and ASPS were investigated. It was found that both ALPS and ASPS were acid protein-bound heteropolysaccharides, although with considerably different chemical composition and molecular weight distribution. Meanwhile, in comparison with ALPS, ASPS exhibited stronger antioxidant activity and inhibitory potential against α-glucosidase according to the *in vitro* evaluation. Moreover, our results suggested that protein and uronic acid might, at least partly, contribute positively to the biological behavior of ALPS and ASPS.

## 1. Introduction

Type 2 Diabetes mellitus (DM), known as non insulin-dependent DM, is a serious metabolic disorder that affects more than 90% of the diabetic population [1]. Hyperglycemia, a condition characterized by an abnormal postprandial increase in blood glucose level, has been linked to the onset of type 2 DM [2]. Meantime, abundant evidence has also suggested that diabetic patients are under oxidative stress, with an imbalance between free radical generating and radical scavenging capacities [3]. Control of postprandial hyperglycemia and inhibition of oxidative stress are suggested to be important in the treatment of type 2 DM [4]. Thus, it has attracted intense interest from researchers in seeking natural antioxidants, including polyphenols and polysaccharides with inhibitory effects on α-glucosidase which is linked to postprandial hyperglycemia, from a wide range of food/medicinal plants [4,5,6,7]. *Ampelopsis grossdentata *[Hand-Mazz] W.T. Wang as a kind of medicinal plant widely distributed in southern China. Its tender leaves and stems have not only been used as a herbal remedy for treating hyperglycemia, hypertension, hepatitis, flu, and sore throats, but also as a daily drink by local people [8,9]. Previous studies mostly focused on flavonoids, like dihydromyricetin and myricetin, which were considered to be the main active components of *Ampelopsis grossdentata*. [9,10,11]. However, little was known about the hypoglycemic potential and antioxidant activities of polysaccharides from *Ampelopsis grossdentata* leaves and stems. Therefore, the objectives of the present study were to analyze the physicochemical properties of the isolated polysaccharides (designated ALPS and ASPS below) from *Ampelopsis grossdentata* leaves and stems, and evaluate their *in vitro* antioxidant activities and inhibitory effects on α-glucosidase.

## 2. Results

### 2.1. Physicochemical Properties of ALPS and ASPS

The yields and chemical composition of ALPS and ASPS are summarized in Table 1. As shown, there was an obvious difference between the main component contents of ALPS and ASPS. 

**Table 1:** Chemical composition of ALPS and ASPS.

Sample	ALPS	ASPS
Yield (%)	1.1 ± 0.1 ^a^	1.0 ± 0.1 ^a^
Protein (%)	3.1 ± 0.3 ^a^	8.8 ± 0.4 ^b^
Neutral sugar (%)	34.7 ± 2.4 ^b^	24.2 ± 1.7 ^b^
Uronic acid (%)	27.5 ± 3.1 ^a^	40.0 ± 2.4 ^b^
Neutral sugar composition (mol ratio)		
L-rhamnose	0.6	1.3
D-fucose	0.2	0.2
L-arabinose	2.5	2.2
D-xylose	0.1	0.3
D-mannose	0.8	3.7
D-glucose	1.6	0.8
D-galactose	4.2	1.6

^a, b^ Means with the same letter are not significantly different (*p* > 0.05).

Compared with ASPS, ALPS had higher (*p* < 0.05) content of neutral sugar, but lower (*p* < 0.05) contents of protein and uronic acid. Monosaccharide composition analysis demonstrated that both polysaccharides were composed of seven monosaccharides, but with different molar ratios (Table 1). D-Galactose, L-arabinose and D-glucose were the dominant monosaccharides in ALPS, while the dominant monosaccharides in ASPS were D-mannose, L-arabinose and D-mannose. The HPGPC records of ALPS and ASPS displayed two and three distinct peaks, and the molecular weight distribution of ALPS and ASPS ranged from 4.6 × 10^3^ to 6.2 × 10^5^ Da and from 3.2 × 10^4^ to 6.1 × 10^6^ Da, respectively. These results corroborated that both ALPS and ASPS were acid protein-bound hetero-polysaccharides. The characteristic IR absorptions of ALPS and ASPS further confirmed the results of the above chemical analysis, but the IR spectra of ALPS and ASPS were similar and no obvious differences could be observed.

### 2.2. Antioxidant Activities of ALPS and ASPS

#### 2.2.1. DPPH Assay

**Figure 1:**
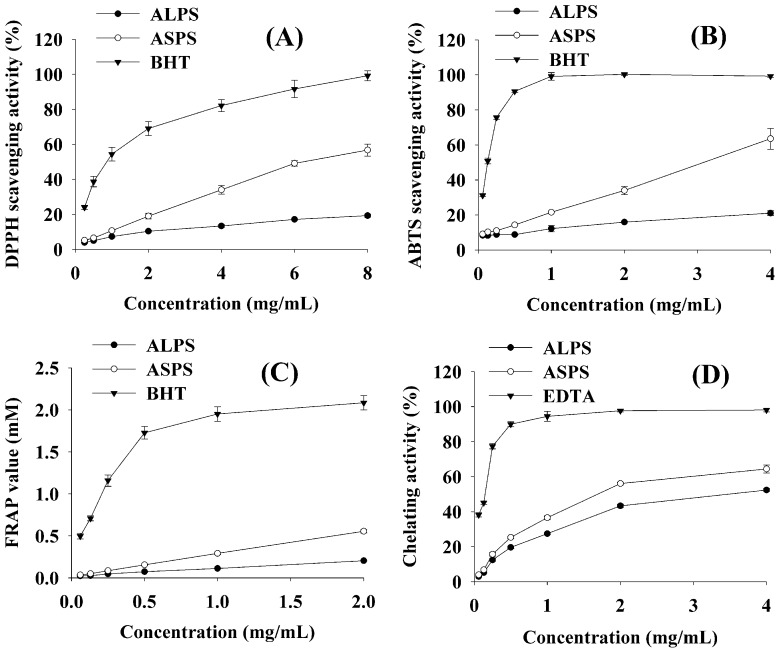
Antioxidant activities of ALPS and ASPS evaluated by the DPPH (**A**), ABTS (**B**), FRAP (**C**) and FIC (**D**) assays.

The DPPH free radical is capable of accepting an electron or a hydrogen radical to become a stable diamagnetic molecule [12], and it has been widely used to test the free radical scavenging ability of various natural products [13,14]. The scavenging ability of ALPS and ASPS on DPPH free radical is shown in Figure 1A. It can be seen that both the polysaccharides and the BHT control exhibited effective concentration-dependent DPPH radical scavenging ability. In the concentrations range from 0.25 to 8 mg/mL, the scavenging effect of BHT ranged from 24% to 99.1%, meanwhile, that of ALPS from 3.9% to 19.3% and that of ASPS from 5.1% to 56.7%. In the tested concentration range, the order of scavenging ability was BHT > ASPS > ALPS. 

#### 2.2.2. ABTS Assay

ABTS cation radicals are more reactive than DPPH radicals and unlike the reactions with the latter, which involve H atom transfer, the reactions with ABTS cation radicals involve an electron transfer process [15]. The scavenging ability of ALPS and ASPS on ABTS cation radicals is shown in Figure 1B. The scavenging effect of ASPS increased faster as the concentration increased than that of ALPS. At concentrations from 0.06 to 4 mg/mL, the scavenging effect of BHT ranged from 31.2% to 99.3%, while, that of ALPS from 8.4% to 21.1% and that of ASPS from 9.1% to 63.5%, so the scavenging effect decreased in the order of BHT > ASPS > ALPS.

#### 2.2.3. FRAP Assay

FRAP measures the antioxidant effect of any substance in the reaction medium as reducing ability, which represents the ability of an antioxidant to donate electrons [16]. As shown in Figure 1C, compared with the positive control both ALPS and ASPS were not good at reducing TPTZ-Fe (III) complex to TPTZ-Fe (II) complex, as. ALPS exhibited a slight increase of FRAP values as the concentrations increased from 0.06 to 2 mg/mL, while, the FRAP values of BHT ranged from 0.5 mM to 2.1 mM, and that of ASPS from 0.03 mM to 0.6 mM, indicating that the ferric-reducing power decreased in the following order: BHT > ASPS > ALPS. 

#### 2.2.4. FIC Assay

It is well-known that ferrous chelating ability may be involved in antioxidant activity and affect other functions which contribute to antioxidant activity [17]. As shown in Figure 1D, the ferrous ion chelating activity of ALPS, ASPS and EDTA increased with increasing concentration. In the tested range from 0.06 to 4 mg/mL, the ferrous ion chelating effect of EDTA ranged from 38.2% to 98.1%, and that of ALPS from 5.2% to 52.4% and that of ASPS from 4% to 64.4%. The ferrous ion chelating activity thus decreased in the following order: EDTA > ASPS > ALPS. 

### 2.3. Inhibitory Activity on α-Glucosidase of ALPS and ASPS

**Figure 2:**
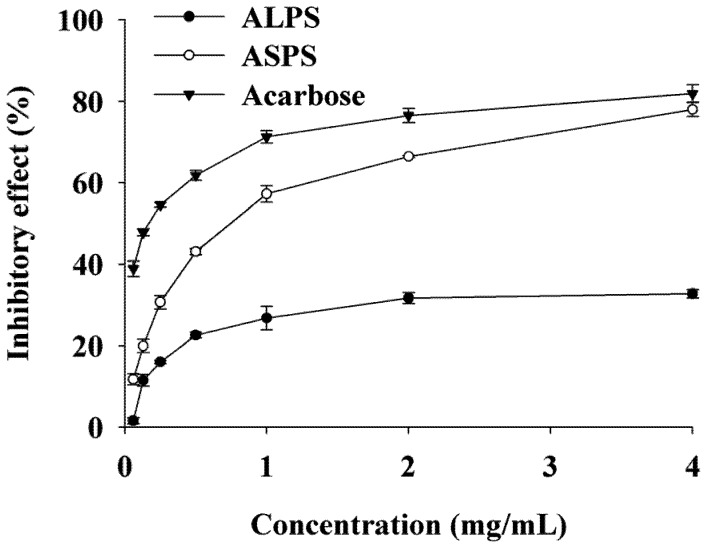
Inhibitory effects of ALPS and ASPS on α-glucosidase.

The inhibitory effects of ALPS and ASPS on α-glucosidase are shown in Figure 2.

It can be observed that both the two polysaccharides and acarbose showed a dose-dependent inhibition effect on α-glucosidase. Obviously, ASPS presented stronger inhibition potential than ALPS in the concentration range from 0.06 to 4 mg/mL. Furthermore, at a concentration of 4 mg/mL, the inhibition effect of ASPS on α-glucosidase reached 78%, which was not significantly different (*p *> 0.05) from that of acarbose (81.9%), but over 1.3-fold higher than that of ALPS (32.8%).

## 3. Discussion

A vast number of studies have revealed that oxidative stress is associated with a wide range of diseases [18,19]. Thus, the antioxidant activity of bioactive compounds from various sources can be considered as an important index by which their potential benefits for human health can be evaluated. In our present study, the antioxidant activities of ALPS and ASPS were assessed by the DPPH, ABTS, FRAP and FIC assays, respectively. Despite the fact the different reaction mechanisms of these antioxidant assays may affect the performance of the two polysaccharides, the results clearly showed that ASPS possessed much stronger antioxidant activity than ALPS (Figures 1A-D The antioxidant activity of plant polysaccharides may be influenced by lots of factors, such as structure, MW and chemical composition [20]. For example, hydroxyl groups of monosaccharides in a polysaccharide were considered to be a significant factor in affecting free radical scavenging ability due to their potential to donate hydrogen [21]. Simple polysaccharides are not supposed to participate in electron transfer reactions with ABTS, however the scavenging activity of polysaccharides from various plant sources on ABTS cation radicals has been reported previously [22,23,24], which may been explained by the different chemical components of the crude polysaccharides, like protein and uronic acid. It was found that higher contents of protein and uronic acid in polysaccharides leaded to higher antioxidant activity [4,25], but no consensus has been reached on this subject since the chemical composition and structure of polysaccharides from various sources is complex [24]. In our study, the protein and uronic acid contents in ASPS were much higher (*p* < 0.05) than those in ALPS (Table 1). According to the antioxidant activity assay results, it is reasonable to infer that protein and uronic acid may have positive effects on the antioxidant activity of the two polysaccharides, although that needs further confirmation by comparative studies on the crude and purified polysaccharides. In addition, MW could also play a role in antioxidant ability because polysaccharides with lower MW exhibited more effective antioxidant capacity than those with higher MW [20], although it seems difficult to figure out the actual role this could have played in the antioxidant activities of the two polysaccharides in our study due to its non-regular distribution in both ASPS and ALPS.

α-Glucosidase, a key enzyme for carbohydrate digestion, has been recognized as a therapeutic target for modulation of postprandial hyperglycemia, which is the earliest metabolic abnormality to occur in type 2 DM [26]. Some kinds of polysaccharides from food/medicinal plants have been found to have inhibitory effect on α-glucosidase [4,7,27], but until now there have been no reported studies on the effects of *Ampelopsis grossedentata* polysaccharides on the activity of α-glucosidase. In this study, ASPS exhibited remarkable inhibitory potential against α-glucosidase, which was much stronger than that of ALPS (Figure 2), indicating that the inhibitory effect on α-glucosidase of ASPS could be one of the mechanisms for the hypoglycemic activity of *Ampelopsis grossedentata*. However, the underlying mechanisms of inhibition on α-glucosidase of polysaccharides are not well known yet. 

Lots of evidence suggests that diabetic patients were under oxidative stress, with an imbalance between the free-radical-generating and radical-scavenging capacities [3,19]. The increased free radical production and reduced antioxidant defense may partially mediate the initiation and progression of diabetes-associated complications [28]. Our results suggested that compared with ALPS, ASPS possessed higher antioxidant activity and stronger inhibitory potential against α-glucosidase, which are important for management of postprandial hyperglycemia. However, the bioavailability of the polysaccharide may be limited due to its high MW. Thus, the hypoglycemic effect of ASPS needs to be validatedin *in vivo* models.

## 4. Experimental

### 4.1. Materials and Chemicals

The leaves and stems of *Ampelopsis grossedentata *were obtained from Sanming in Fujian Province, China. Dialysis membrane (7,000 Da), galacturonic acid, dextran, acarbose, butylated hydroxytoluene (BHT), 1,1-diphenyl-2-picrylhydrazyl (DPPH), 2,2’-azino-bis(3-ethylbenzothiazoline-6-sulphonic acid) diammonium salt (ABTS), 3-(2-pyridyl)-5,6-diphenyl-1,2,4-triazine-4’,4”-disulfonic acid sodium salt (ferrozine), 2,4,6-tripyridyl-s-triazine (TPTZ), L-rhamnose, D-fucose, L-arabinose, D-xylose, D-mannose, D-glucose, D-galactose and baker’s yeast α-glucosidase (EC 3.2.1.20) were obtained from Sigma Chemical Co. (St Louis, MO, USA). Coomassie brilliant blue G-250 and bovine serum albumin were purchased from Sinopharm Chemical Reagent Co. (Shanghai, China). *p*-Nitrophenyl-α-D-glucopyranoside (*p*NPG) was obtained from Xibao Co. (Shanghai, China). All other chemicals were analytical grade and purchased from Shanghai Boer Chemical Reagent Co. (Shanghai, China). 

### 4.2. Extraction and Isolation of ALPS and ASPS

ALPS and ASPS were obtained following the procedure of Chen *et al.* [4]. Briefly, dry meshed *Ampelopsis grossedentata* leaves and stems powders (100 g) were mixed with ethanol-water (1 L, 80:20, v/v) for 24 h to remove most polyphenols and monosaccharide. After the supernatant was removed, the residue was dried in air and then extracted with hot water at 70 °C for 60 min (three times). The aqueous extract was concentrated and then precipitated with a 4-fold volume of 95% ethanol. The precipitate that formed was collected by centrifugation at 3,000×g for 10 min and repeatedly washed sequentially with ethanol, acetone, and ether, respectively for 3 times. The precipitate was dissolved in hot water (70 °C) and excluded protein by the Sevag method, and dialyzed against distilled water for 48 h with dialysis tubing (molecular weight cut-off 7,000 Da) to remove low-molecular weight matter, and then concentrated and precipitated with a 4-fold volume of 95% ethanol to obtain the polysaccharide-enriched fraction. This fraction was dissolved in water (60 °C) to remove the rest of ethanol in a rotary evaporator under reduced pressure, and lyophilized to finally yield ALPS and ASPS.

### 4.3. Chemical Component Analysis

Neutral sugar content was measured by the anthrone-sulfuric acid method [29], using D-glucose as the standard. Uronic acid content was determined by the carbazole-sulfuric acid method using galacturonic acid as the standard [30]. Protein was analyzed by the method of Bradford [31] using bovine serum albumin as the standard.

### 4.4. Monosaccharide Composition Analysis

ALPS and ASPS were hydrolyzed respectively in 2 M trifluoroacetic acid at 120 °C for 2 h. The hydrolysate was converted into its respective alditol acetate by reduction with NaBH_4_ and acetylation with acetic anhydride and analyzed on a Agilent-6890 GC (Waldbronn, Germany) equipped with an Elite-17 ms column (30 m × 0.32 mm × 0.25 µm) and a flame-ionization detector (FID). The temperatures of injector and detector were 250 °C and 230 °C, respectively. The column temperature program was set to hold for 3 min at 190 °C, then to increase to 230 °C at 4 °C /min. N_2_ was used as carrier gas at a flow rate of 1.2 mL/min. The monosaccharide standards (L-rhamnose, D-fucose, L-arabinose, D-xylose, D-mannose, D-glucose and D-galactose) were measured following the same procedure with myo-inositol as the internal standard. 

### 4.5. Molecular Weight (MW) Determination

The MW of ALPS and ASPS was determined by high performance gel permeation chromatography (HPGPC) equipped with a Waters 515 chromatography, a TOSOH BIOSEP G4000SWXL column (7.8 × 300 mm, Tokyo, Japan) and a Waters 2410 RI detector. Fifty µL of the samples were injected and eluted at a buffer (0.1 M NaNO_3_ solution) flow rate of 0.8 mL/min at 40 °C. The HPGPC system was precalibrated with various standard dextrans of different molecular weights (10.5 kDa, 43.2 kDa, 76.9 kDa, 473 kDa and 2,000 kDa).

### 4.6. Infrared Spectral Analysis

The IR spectra of ALPS and ASPS were determined using an Avatar 370 Fourier Transform Infrared (FTIR) spectrophotometer (Thermo Nicolet, Madison, WI, USA). The polysaccharides were ground with KBr powder and then pressed into pellets for FTIR measurement in a frequency range of 4,000–400 cm^−1^.

### 4.7. Antioxidant Activities Determination

#### 4.7.1. DPPH Assay

The DPPH free radical scavenging activity of ALPS and ASPS was determined by the method of Mohsen and Ammar [32], with slight modification. One mL of the tested samples at various concentrations (0.25–8 mg/mL) was added to ethanolic DPPH solution (3 mL, 0.1 mM). Discoloration was measured at 517 nm after incubation for 30 min at 30 °C in the dark. BHT was used as the positive control. The DPPH scavenging effect was calculated as follows:

DPPH scavenging effect (%) = [1 – (A _samp__le_ – A _blank_) / A _cont__rol_] *×*100 (1)

where A _samp__le_, A _blank_ and A _cont__rol_ were defined as absorbance of sample and DPPH, sample without DPPH, and DPPH without sample, respectively.

#### 4.7.2. ABTS Assay

ABTS assay was carried out according to the method of Cai *et al.* [33]. The ABTS cation radical solution was prepared by mixing 7 mM ABTS and 2.45 mM potassium persulphate and incubating in the dark at room temperature for 12 h. The ABTS cation radical solution was then diluted with water to obtain an absorbance of 0.70 ± 0.02 at 734 nm. ABTS cation radical solution (3 mL) was added to the test samples (0.1 mL) of various concentrations (0.06–4 mg/mL) and mixed vigorously. The absorbance was measured at 734 nm after standing for 6 min. BHT was used as the positive control. The ABTS scavenging effect was calculated as follows:

ABTS scavenging effect (%) = [1 – (A _samp__le_ – A _blank_) / A _cont__rol_] *×*100 (2)

where A _samp__le_, A _blank_ and A _cont__rol_ were defined as absorbance of sample and ABTS, sample without ABTS, and ABTS without sample, respectively.

#### 4.7.3. Ferric-Reducing Antioxidant Power (FRAP) Assay

The FRAP assay was performed according to a modified method of Benzie and Strain [34]. Briefly, the working FRAP reagent was prepared by mixing 10 vol of 300 mM acetate buffer (pH 3.6) with TPTZ (100 mL, 10 mM) in HCl (40 mM) and with FeCl_3_ (100 mL, 20 mM). Freshly prepared FRAP reagent was warmed at 37 °C, and a reagent blank reading was taken at 593 nm. Subsequently, test samples (0.6 mL) were added to the FRAP reagent (4.5 mL). A second reading at 593 nm was performed after 8 min. The initial blank reading with the FRAP reagent alone was subtracted from the final reading of the FRAP reagent with the sample to determine the FRAP value of the sample. A standard curve was prepared using different concentrations (0.05–2.5 mM) of FeSO_4_·7H_2_O. BHT was used as the positive control. The reducing ability was expressed as the equivalent to that of 1 mM FeSO_4_·7H_2_O.

#### 4.7.4. Ferrous Ion Chelating (FIC) Assay

The chelating effect of ALPS and ASPS on ferrous ion was assayed according to Yuan *et al.* [35] with a few modifications. One mL of the test samples of various concentrations (0.06–4 mg/mL) was mixed with FeSO_4_ (1 mL, 0.1 mM) for 30 s, then ferrozine (1 mL, 0.25 mM) was added and the mixture was kept for 10 min at room temperature. The absorbance was measured at 734 nm after standing for 6 min. BHT was used as the positive control. The chelating effect on ferrous ion was calculated as follows:

Chelating effect (%) = [1 – (A _samp__le_ – A _blank_) / A _cont__rol_] *× *100 (3)

where A _samp__le_, A _blank_ and A _cont__rol_ were defined as absorbance of sample and ferrozine, sample without ferrozine, and ferrozine without sample, respectively.

### 4.8. α-Glucosidase Inhibition Assay

The inhibitory activity on α-glucosidase of ALPS and ASPS was determined according to the method described by Apostolidis and Lee [1] with a slight modification. A mixture of sample (50 μL) and 0.1 M phosphate buffer (pH 6.9, 100 μL) containing α-glucosidase solution (1 U/mL) was incubated in 96 well plates at 25 °C for 10 min. After preincubation, 5 mM *p*NPG solution (50 μL) in 0.1 M phosphate buffer (pH 6.9) was added to each well at timed intervals. The reaction mixtures were incubated at 25 °C for 5 min. Before and after incubation, absorbance was recorded at 405 nm by microplate reader (SpectraMax M5, Molecular Devices, Sunnyvale, CA, USA). Acarbose was used as the positive control. The inhibitory activity on α-glucosidase was expressed as inhibition percent and was calculated as follows:

Inhibition (%) = [1 – (A _sample_ – A _blank_) / A _control_] *× *100 (4)

where A _samp__le_, A _blank_ and A _cont__rol_ were defined as absorbance of sample and α-glucosidase solution, sample without α-glucosidase solution, and α-glucosidase solution without sample, respectively.

### 4.9. Statistical Analysis

All the experiments were carried out in triplicate. The results were expressed as means ± SD and evaluated by analysis of variance (ANOVA) followed by Turkey’s studentized range test carried out on the SAS system for windows V9 (Version 9.1, SAS Institute Inc., Cary, NC, USA), and *p* < 0.05 was regarded as statistically significant.

## 5. Conclusions

Based on the results obtained, it could be concluded that both ALPS and ASPS are acid protein-bound heteropolysaccharides. ASPS had stronger antioxidant activity and α-glucosidase inhibitory potential in comparison with ALPS, according to *in vitro* evaluation. Moreover, protein and uronic acid are suggested, at least partly, to have positive effects on the biological behavior of ALPS and ASPS. Further studies on the purified ASPS should be carried out to help illustrate the structure-activity relationship of the polysaccharides clearly.

## References

[1] Apostolidis E., Lee C. (2010). *In vitro* potential of *Ascophyllum nodosum* phenolic antioxidant-mediated α-glucosidase and α-amylase inhibition. J. Food Sci..

[2] Haffner S.M. (1998). The importance of hyperglycemia in the nonfasting state to the development of cardiovascular disease. Endocr. Rev..

[3] Yao Y., Sang W., Zhou M., Ren G. (2009). Antioxidant and α-glucosidase inhibitory activity of colored grains in china. J. Agric. Food Chem..

[4] Chen H., Qu Z., Fu L., Dong P., Zhang X. (2009). Physicochemical properties and antioxidant capacity of 3 polysaccharides from green tea, oolong tea, and black tea. J. Food Sci..

[5] Matsui T., Ogunwande I., Abesundara K., Matsumoto K. (2006). Anti-hyperglycemic potential of natural products. Mini-Rev. Med. Chem..

[6] Kwon Y.I., Apostolidis E., Shetty K. (2008). *In vitro* studies of eggplant (*Solanum melongena*) phenolics as inhibitors of key enzymes relevant for type 2 diabetes and hypertension. Bioresour. Technol..

[7] Chen H., Lu X., Qu Z., Wang Z., Zhang L. (2010). Glycosidase inhibitory activity and antioxidant properties of a polysaccharide from the mushroom *Inonotus obliquus*. J. Food Biochem..

[8] Cheng Y. (2004). Research Development of *Ampelopsis grossedentata*. Econ. Forest Res..

[9] Gao J., Liu B., Ning Z., Zhao R., Zhang A., Wu Q. (2009). Characterization and antioxidant activity of flavonoid-rich extracts from leaves of *Ampelopsis grossedentata*. J. Food Biochem..

[10] Zhang Y., Ning Z., Yang S., Wu H. (2003). Antioxidation properties and mechanism of action of dihydromyricetin from *Ampelopsis grossedentata*. Acta Pharm. Sinica..

[11] Zhang Y., Zhang Q., Li L., Wang B., Zhao Y., Guo D. (2007). Simultaneous determination and pharmacokinetic studies of dihydromyricetin and myricetin in rat plasma by HPLC-DAD after oral administration of *Ampelopsis grossedentata* decoction. J. Chromatogr. B.

[12] Soare J.R., Dinis T.C.P., Cunha A.P., Almeida L. (1997). Antioxidant activities of some extracts of *Thymus zygis*. Free Radical Res..

[13] Giovanelli G., Buratti S. (2009). Comparison of polyphenolic composition and antioxidant activity of wild Italian blueberries and some cultivated varieties. Food Chem..

[14] Kuda T., Ikemori T. (2009). Minerals, polysaccharides and antioxidant properties of aqueous solutions obtained from macroalgal beach-casts in the Noto Peninsula, Ishikawa, Japan. Food Chem..

[15] Kaviarasan S., Naik G., Gangabhagirathi R., Anuradha C., Priyadarsini K. (2007). *In vitro* studies on antiradical and antioxidant activities of fenugreek (*Trigonella foenum graecum*) seeds. Food Chem..

[16] Shi J., Gong J., Liu J., Wu X., Zhang Y. (2009). Antioxidant capacity of extract from edible flowers of *Prunus mume* in China and its active components. LWT-Food Sci. Technol..

[17] Moure A., Dominguez H., Parajo J.C. (2006). Antioxidant properties of ultrafiltration-recovered soy protein fractions from industrial effluents and their hydrolysates. Process Biochem..

[18] Finkel T., Holbrook N.J. (2000). Oxidants, oxidative stress and the biology of ageing. Nature.

[19] Rains J.L., Jain S.K. (2011). Oxidative stress, insulin signaling and diabetes. Free Radical Biol. Med..

[20] Chen H., Zhang M., Qu Z., Xie B. (2008). Antioxidant activities of different fractions of polysaccharide conjugates from green tea (*Camellia Sinensis*). Food Chem..

[21] Tian L., Zhao Y., Guo C., Yang X. (2010). A comparative study on the antioxidant activities of an acidic polysaccharide and various solvent extracts derived from herbal *Houttuynia cordata*. Carbohydr. Polym..

[22] Luo A.X., He X.J., Zhou S.D., Fan Y.J., Luo A.S., Chun Z. (2010). Purification, composition analysis and antioxidant activity of the polysaccharides from *Dendrobium nobile* Lindl. Carbohydr. Polym..

[23] Ananthi S., Raghavendran H.R., Sunil A.G., Gayathri V., Ramakrishnan G., Vasanthi H.R. (2010). *In vitro* antioxidant and *in vivo* anti-inflammatory potential of crude polysaccharide from *Turbinaria ornata* (Marine Brown Alga). Food Chem. Toxicol..

[24] Lin C.L., Wang C.C., Chang S.C., Inbaraj B.S., Chen B.H. (2009). Antioxidative activity of polysaccharide fractions isolated from *Lycium barbarum* Linnaeus. Int. J. Biol. Macromol..

[25] Chen H., Zhang M., Xie B. (2004). Quantification of uronic acids in tea polysaccharide conjugates and their antioxidant properties. J. Agric. Food Chem..

[26] Lebovitz H. (1998). Postprandial hyperglycaemic state: importance and consequences. Diabetes Res. Clin. Pract..

[27] Zhang J., Tiller C., Shen J., Wang C., Girouard G.S., Dennis D., Barrow C.J., Miao M., Ewart H.S. (2007). Antidiabetic properties of polysaccharide-and polyphenolic-enriched fractions from the brown seaweed *Ascophyllum nodosum*. Can. J. Physiol. Pharmacol..

[28] Maritim A., Sanders R., Watkins J.B. (2003). Effects of α-lipoic acid on biomarkers of oxidative stress in streptozotocin-induced diabetic rats. J. Nutr. Biochem..

[29] Morris D.L. (1948). Quantitative determination of carbohydrates with Dreywood's anthrone reagent. Science.

[30] Bitter T., Muir H.M. (1962). A modified uronic acid carbazole reaction. Anal. Biochem..

[31] Bradford M.M. (1976). A rapid and sensitive method for the quantitation of microgram quantities of protein utilizing the principle of protein-dye binding. Anal. Biochem..

[32] Mohsen S.M., Ammar A.S.M. (2009). Total phenolic contents and antioxidant activity of corn tassel extracts. Food Chem..

[33] Cai Y., Luo Q., Sun M., Corke H. (2004). Antioxidant activity and phenolic compounds of 112 traditional Chinese medicinal plants associated with anticancer. Life sci..

[34] Benzie I.F.F., Strain J. (1999). Ferric reducing/antioxidant power assay: Direct measure of total antioxidant activity of biological fluids and modified version for simultaneous measurement of total antioxidant power and ascorbic acid concentration. Method. Enzymol..

[35] Yuan J.F., Zhang Z.Q., Fan Z.C., Yang J.X. (2008). Antioxidant effects and cytotoxicity of three purified polysaccharides from *Ligusticum chuanxiong Hort*. Carbohydr Polym..

